# A Social Distance Estimation and Crowd Monitoring System for Surveillance Cameras

**DOI:** 10.3390/s22020418

**Published:** 2022-01-06

**Authors:** Mohammad Al-Sa’d, Serkan Kiranyaz, Iftikhar Ahmad, Christian Sundell, Matti Vakkuri, Moncef Gabbouj

**Affiliations:** 1 Faculty of Information Technology and Communication Sciences, Tampere University, 33720 Tampere, Finland; moncef.gabbouj@tuni.fi; 2 Faculty of Medicine, Clinicum, University of Helsinki, 00014 Helsinki, Finland; 3 Department of Electrical Engineering, Qatar University, Doha, Qatar; mkiranyaz@qu.edu.qa; 4 TietoEVRY Oy, Keilalahdentie 2-4, 02101 Espoo, Finland; iftikhar.ahmad@tietoevry.com (I.A.); christian.sundell@tietoevry.com (C.S.); 5 Haltian Oy, Yrttipellontie 1 D3, 90230 Oulu, Finland; matti.vakkuri@haltian.com

**Keywords:** COVID-19, social distancing, video surveillance, person detection and tracking, pose estimation, crowd monitoring

## Abstract

Social distancing is crucial to restrain the spread of diseases such as COVID-19, but complete adherence to safety guidelines is not guaranteed. Monitoring social distancing through mass surveillance is paramount to develop appropriate mitigation plans and exit strategies. Nevertheless, it is a labor-intensive task that is prone to human error and tainted with plausible breaches of privacy. This paper presents a privacy-preserving adaptive social distance estimation and crowd monitoring solution for camera surveillance systems. We develop a novel person localization strategy through pose estimation, build a privacy-preserving adaptive smoothing and tracking model to mitigate occlusions and noisy/missing measurements, compute inter-personal distances in the real-world coordinates, detect social distance infractions, and identify overcrowded regions in a scene. Performance evaluation is carried out by testing the system’s ability in person detection, localization, density estimation, anomaly recognition, and high-risk areas identification. We compare the proposed system to the latest techniques and examine the performance gain delivered by the localization and smoothing/tracking algorithms. Experimental results indicate a considerable improvement, across different metrics, when utilizing the developed system. In addition, they show its potential and functionality for applications other than social distancing.

## 1. Introduction

The rapid outbreak of the Coronavirus Disease 2019 (COVID-19) has imposed restrictions on people’s movement and daily life [1]. Reducing the spread of the virus mandates constraining social interactions, traveling, and access to public areas and events [1]. These limitations arise to mainly advocate social distancing; the practice of increasing physical space among people to minimize virus transmission [2]. Monitoring and maintaining social distancing is carried out by governmental bodies and agencies using mass surveillance systems and closed-circuit television (CCTV) cameras [3]. Nonetheless, this task is cumbersome and suffers from subjective interpretations and human error due to fatigue; hence, computer vision and machine learning tools are convenient for automation [4]. In addition, they enable crowd behavior to be monitored and anomalies such as congested regions, curfew infractions, and illegal gatherings to be recognized. The widespread of mass surveillance and its integration with Machine Learning is hindered by ethical concerns, including possible breach of privacy and potential abuse [3]. Therefore, privacy-preserving surveillance and Machine Learning solutions are paramount to their ethical adoption and application [5].

The design of vision-based social distance estimation and crowd monitoring system deals with the following challenges [4]: (1) geometry understanding, in terms of ground plane identification and homography estimation; (2) multiple people detection and localization; and (3) statistical/temporal characterization for social distance infractions, e.g., short-term violations are irrelevant. Currently, Machine Learning-based solutions identify social distance infringements using off-the-shelf person detection and tracking models [4]. In general, the models’ performance is conjoined with privacy; they yield high performance by carrying and processing person-specific information to develop robustness against occlusions and missing data [4]. In addition, they localize human subjects via bounding boxes that can be over-sized or incomplete which results in significant distance estimation errors [6]. Therefore, we propose a privacy-preserving adaptive social distance estimation and crowd monitoring system that can be implemented on top of any existing CCTV infrastructure. The main contributions of the paper are as follows: (1) Developing a robust person localization strategy using pose estimation techniques; (2) Forming an adaptive smoothing and tracking paradigm to mitigate the problem of occlusions and missing data without compromising privacy; (3) Designing a real-time privacy-preserving social distance estimation and crowd monitoring solution with potential to cover other application areas and tasks.

The rest of this paper is organized as follows: Section 2 overviews the related work and Section 3 describes our methodologies to build and evaluate the proposed system. Afterwards, we present and discuss the system outcome and performance in Section 4. Finally, Section 5 concludes the paper and suggests topics for future research.

## 2. Related Work

This section reviews state-of-the-art Machine Learning-based social distance estimation and monitoring solutions and summarizes their advantages and limitations. First, we analyze various person detection and localization strategies within the scope of social distancing. After that, we review different approaches to recognize social distancing abnormalities. Finally, we discuss the latest vision-based crowd monitoring techniques.

### 2.1. Person Detection and Localization

Several methods exist in the literature and fall under two main categories: object detectors and pose estimation techniques. The former identifies objects by bounding a box around them, while the latter detects the human joints and connects them resulting in pose estimates [7]. On the one hand, object detectors, such as YOLO models [8], are more general-purpose than pose estimation techniques, but their utility for identifying human subjects may require pruning and/or retraining. In addition, they do not offer further information about the detected objects and their bounding boxes can be over-sized or incomplete [6]. On the other hand, pose estimators are specialized models; hence, they are more suitable to detect people in a scene. Specifically, they account for various body orientations/actions such as standing, sitting, riding, and bending, when compared to object detectors [9]. Moreover, their ability to work in dense crowds was verified in [10,11], which is the very same setting social distance monitoring is dealing with. Nonetheless, pose estimators are computationally more expensive than object detectors and their high entropy output requires further processing [7].

In [12], a visual analysis technique is proposed to quantify and monitor contact tracing for COVID-19. The detection and tracking of human subjects are performed by a YOLO architecture and a Simple Online and Real-time Tracking model, respectively. In addition, each subject is localized by its bounding box bottom mid-point. Similar detection and tracking approaches are proposed in [13,14], but the latter localizes the subjects by their bounding box centroid. The aforementioned solutions, although accurate, are not suitable, because they carry person-specific information which hinders their adoption for privacy-preserving applications. Nonetheless, privacy-preserving techniques are developed in [6,15] to monitor the evolution of social distancing patterns using CCTV cameras. The first work utilizes YOLO-v3 to detect pedestrians and the bounding box centroid for localization. Moreover, the second work explores two-person detectors and one end-to-end model and provides evidence that the latter does not necessarily improve performance and the bounding box bottom mid-point is the best for localization. Many variants of the YOLO model and other neural network architectures are used to detect humans in videos and the bounding box centroid, top left edge, or bottom center, is used for localization [16,17,18,19,20,21,22,23,24,25]. Lastly, the social distancing problem is tackled in [26] using a pose estimation model to detect human subjects in videos and to infer their location using the predicted feet joints. The same approach is employed in [27] to measure inter-personal distances but for still images. This has motivated us to use pose estimation techniques to detect people because they offer rich information about the localized subjects and mitigate the pitfalls of bounding boxes.

### 2.2. Anomaly Recognition

The scope of the social distancing problem defines an anomaly in a surveillance video by the presence of social distance violations [4]. This task requires estimating inter-personal distances among the localized subjects and comparing them to a predefined safety threshold [4]. In [13,15], the localized subjects’ pair-wise distances are calculated in the real-world coordinates and social distance violations are identified by a 2 m safety threshold; however, the problem of occlusion is not tackled in [15]. Furthermore, in [12,18,20,23,24], the localization results are morphed to the real-world top-view coordinates to calculate the pair-wise distances. The social distance violations are identified by 1, 1.8, and 2 m safety distances. However, the reported results focus on the person detection performance and they illustrate identifying infractions by a few qualitative examples. Moreover, the developed systems in [18,20,23,24] do not mitigate the problem of occlusion nor missing detections. This is important because these are major limitations and tracking with privacy preservation is an essential remedy [28]. In [21], a centroid tracking algorithm is used to resolve occlusions [29], pair-wise distances are computed, and violations are identified by a 1.8 m safety threshold. However, the performance evaluation is assessed using a single video with only two people in it. This restricts generalizing the system’s efficacy and its applicability to real-life scenarios. Moreover, inter-personal distances are computed in [6] and the violations are identified at three safety levels; 1, 1.8, and 3.6 m. The study concludes that incomplete or over-sized bounding boxes introduce significant errors to the distance calculation; hence, selecting an appropriate person detector is paramount to the system’s feasibility and success. Finally, in [26], pair-wise distances are approximated through the estimated body joints and social distance infractions are identified by a 2 m threshold.

The reviewed literature shows a discrepancy in the safety distance selection for detecting social distance violations. This inconsistency hinders fair comparisons, but it has motivated us to test the proposed system applicability across a wide-range of safety distances and to utilize various performance measures.

### 2.3. Crowd Monitoring

Crowd monitoring aims to attain a high-level understanding of crowd behavior by processing the scene in a global or local manner [30]. Macroscopic methods such as crowd density, crowd counting, and flow estimation, neglect the local features and focus on the scene as a whole [31,32]. In contrast, microscopic techniques start by detecting individual subjects and then group their statistics to summarize the crowd state [33]. These two approaches are complementary in terms of the efficiency/accuracy trade-off. In other words, macroscopic techniques are efficient in handling high-density crowds, while microscopic methods are accurate for sparse groups [31].

An approach to analyze the crowd and social distancing behavior from UAV captured videos is proposed in [31]. Discrete density maps are generated to classify the crowd state in each aerial frame patch as dense, medium, sparse, or empty. In addition, a microscopic technique is employed to detect, track, and compute inter-personal distances. In [34], crowd counting and action recognition techniques are reviewed in the scope of social distancing. The study suggests that density-based approaches are preferred due to their inherent error suppression in which the contribution of faulty counts or missing detections is insignificant to the long-term-averaged density map. Moreover, pedestrians’ spatial patterns are captured in [6] by long-term occupancy and crowd density maps. The former describes the spatial signature exerted by the subjects in the surveilled scene, while the latter encodes the spatial impression of social distance infractions [35]. Similarly, heatmaps are generated in [13,26,36] to represent the regions in which social distance violations are frequent. These studies demonstrate that short and long-term occupancy/crowd density maps are important to identify high-risk regions in the scene. In addition, they allow a quantification for the pedestrians’ compliance with social distancing guidelines [6].

## 3. Methodology

**Figure 1:**
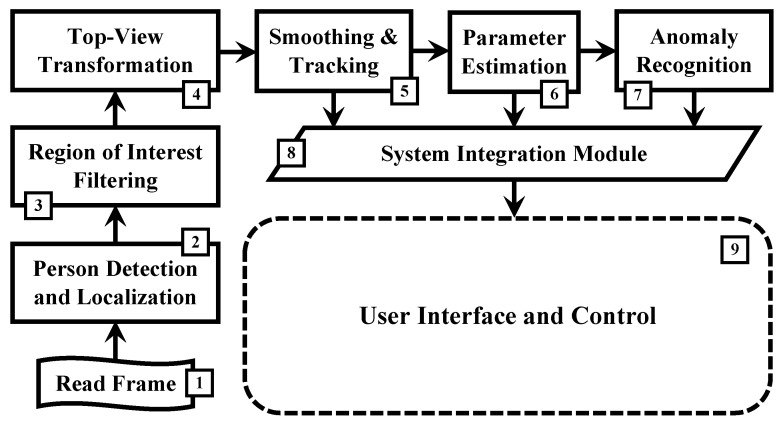
The proposed social distance estimation and crowd monitoring system model. The model is comprised of the following stages: (1) Read a video frame; (2) Detect human subjects and localize their positions; (3) Discard all positions outside the ROI; (4) Transform the remaining positions to the real-world coordinates; (5) Smooth the noisy estimates and compensate for missing data by tracking; (6) Estimate the subjects’ inter-personal distances and crowd density maps; (7) Recognize irregularities in the crowd state in terms of social distance infringements and congestion; (8) Integrate the video frame with the estimated parameters and identified anomalies; and (9) Display the integrated frame and generate a dynamic top-view map for the scene.

The proposed social distance estimation and crowd monitoring system is depicted in Figure 1. The model is comprised of the following stages:

1. Read a frame from the surveillance camera. This component can be adjusted to skip/drop frames in case of using high-resolution and/or high-frame-rate cameras.
2. Detect human subjects in the input frame and compute their position. The position of each detected subject is estimated as a single point.
3. Discard any localized positions outside a selected region of interest (ROI). The ROI is defined by the user beforehand and typically encloses the ground plane.
4. Transform the localized positions from the image–pixel coordinates to the real-world coordinates. This provides a top-view depiction of the subject’s position.
5. Smooth the noisy top-view positions and compensate for missing data due to occlusion with tracking.
6. Estimate the inter-personal distances among the detected subjects and the occupancy/crowd density maps.
7. Recognize social distance violations and identify congested or overcrowded regions in the scene.
8. Integrate the smoothed/tracked positions, estimated parameters, and detected anomalies with the video frame.
9. View the integrated video frame and generate a dynamic top-view map for the scene. This component allows adjusting the type and amount of appended information.

The proposed system design process is governed by the following requirements:

- High accuracy and reliability in terms of robustness to noise and missing data.
- Light weight for implementation and deployment.
- Modularity to facilitate maintenance, upgrades, decentralization, and to avoid resource allocation bottlenecks.
- Privacy-preserving by not carrying nor processing person-specific features.
- Robustness against different vertical pose states and actions, e.g., standing, sitting, bowing, bending, walking, and cycling.

The remaining subsections discuss and detail each stage in the proposed system. We use an example video frame from the EPFL-MPV dataset to illustrate the outcome of each stage—see Section 4.1 for more details on the dataset.

### 3.1. Person Detection and Localization

Given an input video frame, we detect and localize human subjects using a pose estimation technique, because object detection models can yield incomplete or over-sized bounding boxes and they do not offer rich information [6].

#### 3.1.1. Detection

**Figure 2:**
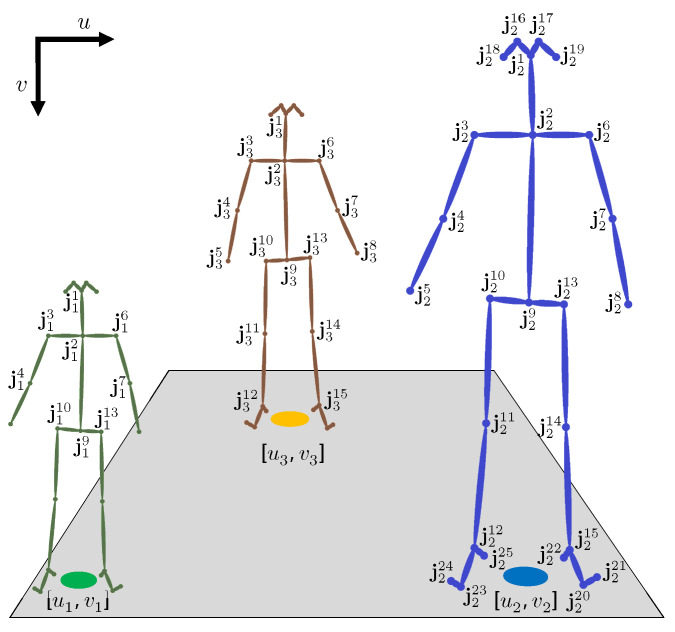
An example pose estimation for three subjects with varying heights and spatial positions. The OpenPose 25 estimated joints are indexed on the right (blue) skeleton. The remaining two subjects have some undetected joints, but their joint indexing remains the same. The ground position of each subject is estimated by the midpoint of their feet joints. The user-defined region of interest is depicted in gray and includes all three ground positions.

We utilize OpenPose to detect and estimate human poses in the input video frame. Specifically, OpenPose estimates and connects the body joints using part affinity fields [37]. Let *N* and *M* be the total number of true and detected subjects in the video frame, respectively, and ${\left\{J_{m}\right\}}_{m\in [1,M]}$ be the set of estimated joints for all detected subjects where $J_{m}={\left\{j_{m}^{j}\right\}}_{j\in [1,25]}$, $j_{m}^{j}=\left[u_{m}^{j},v_{m}^{j}\right]$, $u_{m}^{j}$ and $v_{m}^{j}$ define the horizontal and vertical coordinates of the joint *j*, respectively—see Figure 2 for an example.

Ideally, OpenPose yields 25 joints for each detected subject, but we recognize that some might not be detected due to various reasons. This results in some empty entries in $J_{m}$, but does not change the indexing scheme. Moreover, to model a realistic scenario, we assume that *N* and *M* are not necessarily equal, i.e., the number of detected subjects can be less, equal, or more than the true number of people in a frame. Finally, note that we select OpenPose due to its simplicity and availability, but it can be replaced with any other pose estimation model given the same body joints indexing scheme.

**Figure 3:**
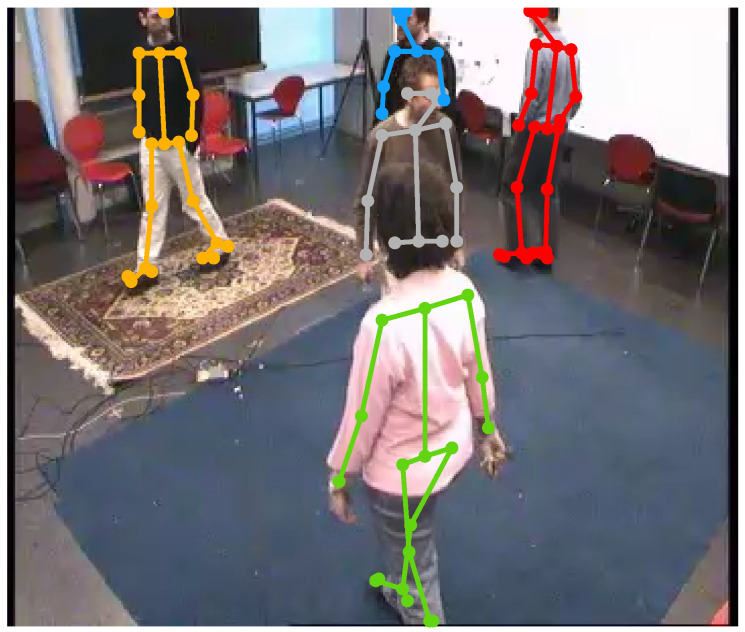
The estimated poses in frame 1824 of the EPFL-MPV dataset scene 6p-c0. Three out of five people are detected correctly (red, orange, and green poses) whereas the rest are not due to partial occlusion and missing data (gray and blue poses).

Figure 3 shows the pose estimation outcome for an example input frame with 5 people moving freely in a room. OpenPose yields five detections shown in gray, red, orange, green, and blue with 13, 22, 20, 17, and 8 total number of connected joints, respectively. The gray and blue poses are incomplete because of partial occlusion and missing data.

#### 3.1.2. Localization

We select the midpoint of the feet of each subject as the anchor to localize their positions, also known as the ground position. The selected point offers reliable estimation because: (1) it is independent of the subjects height, width, and orientation; (2) it lies on the ground; thus, homography transformation is possible; (3) it has a clear definition when compared to bounding boxes; (4) it carries no person-specific information; hence, privacy is preserved.

In [26], given the non-empty set of feet joints $\{j_{m}^{12}$, $j_{m}^{15}$, $j_{m}^{20}$, $j_{m}^{21}$, $j_{m}^{22}$, $j_{m}^{23}$, $j_{m}^{24}$, $j_{m}^{25}\}$ and the condition $\#J_{m}\geq 13$, the ground position of subject *m* is estimated as follows:

(1) $$u_{m}=\left\{\begin{array}{cc} \frac{\sum \left\{u_{m}^{1},u_{m}^{2},u_{m}^{9}\right\}}{\#\left\{u_{m}^{1},u_{m}^{2},u_{m}^{9}\right\}} & :\left\{u_{m}^{1},u_{m}^{2},u_{m}^{9}\right\}\neq \emptyset \\ \frac{\min\left(u_{m}\right)+\max\left(u_{m}\right)}{2} & :\mathrm{otherwise} \end{array}\right.\;,$$

(2) $$v_{m}=\frac{\sum \left\{v_{m}^{12},v_{m}^{15},v_{m}^{20},v_{m}^{21},v_{m}^{22},v_{m}^{23},v_{m}^{24},v_{m}^{25}\right\}}{\#\left\{v_{m}^{12},v_{m}^{15},v_{m}^{20},v_{m}^{21},v_{m}^{22},v_{m}^{23},v_{m}^{24},v_{m}^{25}\right\}}\;,$$

where $u_{m}=\left\{u_{m}^{j}\right\}$, j∈[1,25], and # denotes the number of non-empty elements in the set. We call this approach the basic ground position estimation and argue that it is inadequate because the constraints are quite restrictive. For instance, Equation (1) assumes human subjects with perfect vertical orientation, which may not be the case. In addition, in Equation (2), the sole reliance on detecting any foot joint and the required minimum number of joints limits its applicability in real-life scenarios. In fact, this approach estimates the ground position only when information is abundant. Therefore, we propose a localization strategy that eliminates the basic position pitfalls and relaxes its restrictions and constraints.

Algorithm 1 explains the proposed localization strategy. First, we eliminate the conditions mandated by the basic approach and expand the search space to include the subject’s feet, knees, hips, and torso. In particular, for the horizontal coordinate $u_{m}$, we leverage the joints left/right symmetry by averaging the horizontal position of two opposing joints. For instance, $u_{2}$ and $u_{3}$ in Figure 2 are computed by the 1st case (line 2), while $u_{1}$ is found by the 7th case using the hip joints, i.e., $u_{1}^{10}$ and $u_{1}^{13}$ (line 8). Moreover, for the vertical coordinate $v_{m}$, we relax the requirement for detecting the feet joints by exploiting the human average skeletal characteristics. More specifically, we use the ratio between the torso and lower body lengths to infer the ground position vertical coordinate [26], i.e., (0.85/0.6) in line 15. Finally, regardless of the approach, we discard any estimated positions outside the user-defined ROI—see Figure 2.

**Algorithm 1** The proposed localization strategy.
**Input:**$\left\{u_{m}^{j}\right\}$ and $\left\{v_{m}^{j}\right\}$ where j∈[1,25].
**Output:**$u_{m}$ and $v_{m}$.
**Initialization:** Left/right foot horizontal coordinates α/β and the feet vertical coordinate γ.
$\alpha =\frac{\sum \left\{u_{m}^{15},u_{m}^{20},u_{m}^{21},u_{m}^{22}\right\}}{\#\left\{u_{m}^{15},u_{m}^{20},u_{m}^{21},u_{m}^{22}\right\}}$          $\beta =\frac{\sum \left\{u_{m}^{12},u_{m}^{23},u_{m}^{24},u_{m}^{25}\right\}}{\#\left\{u_{m}^{12},u_{m}^{23},u_{m}^{24},u_{m}^{25}\right\}}$          $\gamma =\{v_{m}^{12}$,$v_{m}^{15}$,$v_{m}^{20}$,$v_{m}^{21}$,$v_{m}^{22}$,$v_{m}^{23}$,$v_{m}^{24}$,$v_{m}^{25}\}$

1:**switch** true **do**

2:    **case** α≠∅∧β≠∅    **then**   $u_{m}=\frac{1}{2}\left(\frac{\sum \alpha }{\#\alpha }+\frac{\sum \beta }{\#\beta }\right)$ and $F_{m}^{u}=1$. 
▹ Both feet joints are available
3:    **case** $\alpha \neq \emptyset \wedge u_{m}^{11}\neq \emptyset$    **then**   $u_{m}=\frac{1}{2}\left(\frac{\sum \alpha }{\#\alpha }+u_{m}^{11}\right)$ and $F_{m}^{u}=2$. 
▹ Left foot and right knee joints are available
4:    **case** $u_{m}^{14}\neq \emptyset \wedge \beta \neq \emptyset$    **then**   $u_{m}=\frac{1}{2}\left(u_{m}^{14}+\frac{\sum \beta }{\#\beta }\right)$ and $F_{m}^{u}=2$. 
▹ Left knee and right foot joints are available
5:    **case** $u_{m}^{11}\neq \emptyset \wedge u_{m}^{14}\neq \emptyset$    **then**   $u_{m}=\frac{1}{2}\left(u_{m}^{11}+u_{m}^{14}\right)$ and $F_{m}^{u}=2$. 
▹ Both knees’ joints are available
6:    **case** $u_{m}^{10}\neq \emptyset \wedge u_{m}^{14}\neq \emptyset$    **then**   $u_{m}=\frac{1}{2}\left(u_{m}^{10}+u_{m}^{14}\right)$ and $F_{m}^{u}=2$. 
▹ Right hip and left knee joints are available
7:    **case** $u_{m}^{11}\neq \emptyset \wedge u_{m}^{13}\neq \emptyset$    **then**   $u_{m}=\frac{1}{2}\left(u_{m}^{11}+u_{m}^{13}\right)$ and $F_{m}^{u}=2$. 
▹ Right knee and left hip joints are available
8:    **case** $u_{m}^{10}\neq \emptyset \wedge u_{m}^{13}\neq \emptyset$    **then**   $u_{m}=\frac{1}{2}\left(u_{m}^{10}+u_{m}^{13}\right)$ and $F_{m}^{u}=2$. 
▹ Hip’s joints are available
9:    **case** $u_{m}^{2}\neq \emptyset \wedge u_{m}^{9}\neq \emptyset$    **then**   $u_{m}=\frac{1}{2}\left(u_{m}^{2}+u_{m}^{9}\right)$ and $F_{m}^{u}=2$. 
▹ Torso’s joints are available
10:    **case** α≠∅∨β≠∅    **then**   $u_{m}=\frac{\sum \left\{\alpha ,\beta \right\}}{\#\left\{\alpha ,\beta \right\}}$ and $F_{m}^{u}=2$. 
▹ Consider any available feet joints
11:    **otherwise**   $u_{m}=\emptyset$ and $F_{m}^{u}=0$.

12:
**end switch**


13:**switch** true **do**

14:    **case** γ≠∅    **then**   $v_{m}=\frac{\sum \gamma }{\#\gamma }$ and $F_{m}^{v}=1$. 
▹ Consider any available feet joints
15:    **case** $v_{m}^{2}\neq \emptyset \wedge v_{m}^{9}\neq \emptyset$    **then**   $v_{m}=v_{m}^{9}+\left(0.85/0.6\right)\left|v_{m}^{2}-v_{m}^{9}\right|$ and $F_{m}^{v}=2$. 
▹ Torso’s joints are available
16:    **otherwise**   $v_{m}=\emptyset$ and $F_{m}^{v}=0$.

17:
**end switch**



The proposed localization strategy is driven by the argument that noisy measurements with known error states are more valuable than no measurements at all. In other words, if we predict the subject’s ground position and supplement it with the state of available information, we can append each prediction with a flag describing its integrity, or confidence level. In this work, we coin this concept by forming the error state flags $F_{m}^{u}$ and $F_{m}^{v}$ in the following manner:

- $F_{m}^{u}=\emptyset$ ($F_{m}^{v}=\emptyset$): subject is not detected.
- $F_{m}^{u}=0$ ($F_{m}^{v}=0$): subject is detected but $u_{m}$ ($v_{m}$) is not available, regardless of the reason.
- $F_{m}^{u}=1$ ($F_{m}^{v}=1$): subject is detected and $u_{m}$ ($v_{m}$) is directly estimated from the feet joints.
- $F_{m}^{u}=2$ ($F_{m}^{v}=2$): subject is detected and $u_{m}$ ($v_{m}$) is predicted using other joints.

Similarly, an overall localization error flag is constructed for each detected subject *m* as follows:

(3) $$F_{m}=\left\{\begin{array}{c} \emptyset :F_{m}^{u}=\emptyset \vee F_{m}^{v}=\emptyset \\ 0:F_{m}^{u}=0\vee F_{m}^{v}=0\\ 1:F_{m}^{u}=1\wedge F_{m}^{v}=1\\ 2:F_{m}^{u}=2\vee F_{m}^{v}=2 \end{array}\right.\;.$$

**Figure 4:**
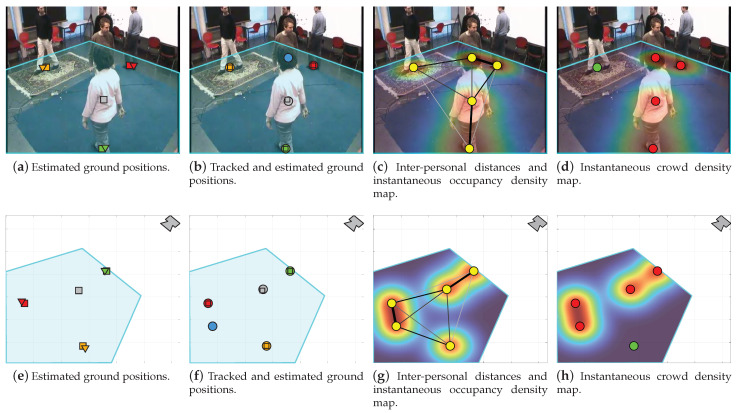
The proposed system outcome at each stage using the example input frame and the estimated poses in Figure 3. (**a**–**d**) demonstrate the localized human subjects, smoothed/tracked ground positions, inter-personal distances with the instantaneous occupancy map, and the instantaneous crowd map along with the detected social distance violations in the image–pixel coordinates, respectively. (**e**–**h**) present the same results as in (**a**–**d**), but in the real-world coordinates. The user-selected ROI is shown in cyan and covers the floor plane in the scene. The basic and proposed localization results are depicted by triangles and squares, respectively, while the smoothed/tracked ground positions are visualized with circles. The distances among the subjects are visualized using lines with varying thickness and darkness where thick/thin and dark/light lines indicate shorter/longer distances. The instantaneous occupancy and crowd density maps are computed with a 1 m spatial resolution (δ=1) and 2 m social safety distance (r=2), respectively. Note that the ground positions in (**a**,**b**,**e**,**f**) are color-coded in accordance with the estimated poses in Figure 3. The color notion is dropped in (**c**,**d**,**g**,**h**) to preserve privacy and to emphasize the recognition of a social distance infringement; red/green indicates the presence/absence of subjects violating the defined social safety distance.

Figure 4a demonstrates the basic and proposed localization results using the estimated poses in Figure 3. In addition, it shows the selected ROI in cyan, which encloses the floor plane in the scene. By examining Figure 4a, one notes that both localization strategies yield valid estimates when supplied with enough number of connected joints. However, the proposed approach is more accurate since it does not assume perfect vertical orientation. Moreover, it mitigates partial occlusion by inferring the position vertical coordinate using the torso to lower body lengths ratio—see the estimated position in gray. Nonetheless, both strategies are limited, because they cannot resolve the ground position when information is scarce or completely missing. For instance, they cannot localize the fifth subject, the one with the blue pose in Figure 3 because we only have a few joints.

### 3.2. Top-View Transformation

Let us assume that the surveillance camera is placed at height *h* and oriented with a pan and tilt angles $\theta_{p}$ and $\theta_{t}$, respectively. The transformation from a three-dimensional position in the real-world coordinates to its corresponding two-dimensional (2D) position in the image–pixel coordinates; [x,y,z]→[u,v] is expressed as follows:

(4) $$\left[\begin{array}{c} u\\ v\\ 1 \end{array}\right]=\frac{1}{\alpha_{s}}\;K\left[R\;\;\;T_{0}\right]\left[\begin{array}{c} x\\ y\\ z\\ 1 \end{array}\right]\;,$$

where $\alpha_{s}$ is the image-to-real distance scale, $K\in R^{3\times 3}$ is the camera intrinsic parameter matrix which maps the camera coordinates to the image coordinates, $\left[R\;\;\;T_{0}\right]$ maps the real-world coordinates to the camera coordinates, $R\in R^{3\times 3}$ is a rotation matrix that compensates for the camera orientation ($\theta_{p}$ and $\theta_{t}$), and $T_{0}\in R^{3\times 1}$ is a translation vector which deals with the camera position and height. Since we are only concerned with transforming the subjects’ ground positions from the image coordinates $\left[u_{m},v_{m}\right]$ to the real-world ground plane $\left[x_{m},y_{m}\right]$, Equation (4) simplifies to:

(5) $$\left[\begin{array}{c} x_{m}\\ y_{m}\\ 1 \end{array}\right]=\alpha_{s}\;H^{-1}\left[\begin{array}{c} u_{m}\\ v_{m}\\ 1 \end{array}\right]\;,$$

where $H\in R^{3\times 3}$ is the camera homography matrix. This transformation results in a top-view depiction of the subject’s real-world positions—see Figure 4e.

In this work, we assume the homography matrix *H* and the image-to-real distance scale $\alpha_{s}$ to be known for simplicity; however, they can be obtained by GPS and accelerometers [38,39,40], determined by calibration [41,42], inferred from the computed poses [26,27], or estimated by a four-point perspective transformation [43].

### 3.3. Smoothing and Tracking

The top-view transformed ground positions are noisy and suffer from missing values. The former is due to uncertainties and errors in the localization technique while the latter comes from occlusions. In this section, we formulate the estimated positions temporal evolution by a constant velocity model. Afterwards, we compensate for localization errors and missing measurements by a linear Kalman filter (KF) and a global nearest neighbor (GNN) tracker.

#### 3.3.1. State and Measurement Models

Let $x_{m,t}={\left[x_{m,t},{\dot{x}}_{m,t},y_{m,t},{\dot{y}}_{m,t}\right]}^{T}$ be the state vector of subject *m* that defines its ground position and velocity at frame *t*. Assuming constant velocity, $x_{m,t}$ and its measured counterpart $y_{m,t}$ are expressed as follows [44]:

(6) $$x_{m,t}=F\;x_{m,t-1}+\omega_{m,t-1}\;,$$

(7) $$y_{m,t}=H\;x_{m,t}+\nu_{m,t}\;,$$

where F is a constant state transition matrix from $x_{m,t-1}$ to $x_{m,t}$, H is a constant state-to-measurement matrix, $\omega_{m,t}$∼$N(0,Q_{m,t})$, and $\nu_{m,t}$∼$N(0,R_{m,t})$.

#### 3.3.2. The Linear Kalman Filter

**Figure 5:**
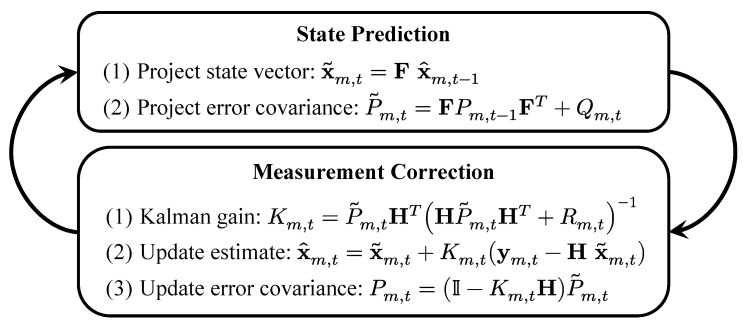
The linear Kalman filter process is comprised of two stages; state prediction and measurement correction.

The KF offers an optimal estimate for $x_{m,t}$ given the measurement $y_{m,t}$ by following the process depicted in Figure 5. First, given a previous (or initial) posterior estimate ${\hat{x}}_{m,t-1}$ with error covariance $P_{m,t-1}$, the KF predicts a prior estimate ${\tilde{x}}_{m,t}$ and computes its error covariance ${\tilde{P}}_{m,t}$. Afterwards, it calculates the posterior estimate ${\hat{x}}_{m,t}$ with error covariance $P_{m,t}$ using a Kalman filter gain $K_{m,t}$. Finally, the process repeats using ${\hat{x}}_{m,t}$ and $P_{m,t}$ as inputs to the state prediction stage.

By examining the Kalman gain equation in the measurement correction stage in Figure 5, one notes that increasing/decreasing $R_{m,t}$ decreases/increases the reliance of ${\hat{x}}_{m,t}$ on the measurement $y_{m,t}$. In this work, we control this mechanism by adjusting the variance $\sigma_{m,t}^{2}$ in $R_{m,t}$ according to the overall localization error flag $F_{m,t}$, i.e., [45]:

(8) $$\sigma_{m,t}^{2}=\left\{\begin{array}{cc} \sigma_{1}^{2} & :F_{m,t}=0\\ \sigma_{2}^{2} & :F_{m,t}=1\\ \sigma_{3}^{2} & :F_{m,t}=2 \end{array}\right.\;.$$

In other words, the measurement error variance is adapted to smooth the estimated positions according to their appended quality. Consequently, the KF reduces the localization noise and can offer posterior estimates when the measurement is missing [45]. Nevertheless, the KF equations require knowing the correspondence between the detections/predictions at consecutive frames. This is generally tackled via multiple object tracking (MOT) approaches such as the global nearest neighbor (GNN) algorithm.

#### 3.3.3. Global Nearest Neighbor Tracking

GNN is a real-time light-weight MOT solution that tracks objects by assigning detections/predictions to tracks, and by maintaining its track record [46]. It solves the assignment task by minimizing the following cost function:

(9) $$\min_{\alpha_{m,q}}\left[\sum_{m=1}^{M}\sum_{q=1}^{Q}C_{m,q}\;\alpha_{m,q}\right]\;s.t.\;\sum_{m=1}^{M}\alpha_{m,q}=1\;\;\forall q\;\mathrm{and}\;\sum_{q=1}^{Q}\alpha_{m,q}=1\;\;\forall m\;,$$

where *M* is the number of detected subjects, *Q* is the number of maintained (or initiated) tracks, $C_{m,q}$ is the cost of assigning detection *m* to track *q* and $\alpha_{m,q}\in \left\{0,1\right\}$ such that if detection *m* is assigned to track *q*, then $\alpha_{m,q}=1$, otherwise $\alpha_{m,q}=0$. The constraints in Equation (9) ensure that each detection can be assigned to only one track and vice versa.

The GNN defines the assignment cost $C_{m,q}$ in Equation (9) as follows:

(10) $$C_{m,q}=D\left(y_{m,t},{\hat{y}}_{q,t}\right)+\log\left|HP_{q,t}H^{T}+R_{q,t}\right|\;,$$

(11) $$D^{2}\left(y_{m,t},{\hat{y}}_{q,t}\right)={\left(y_{m,t}-{\hat{y}}_{q,t}\right)}^{T}{\left(HP_{m,t}H^{T}+R_{m,t}\right)}^{-1}\left(y_{m,t}-{\hat{y}}_{q,t}\right)\leq \gamma_{g}\;,$$

where ${\hat{y}}_{q,t}=H\;{\hat{x}}_{q,t}$ is the estimated measurement with error covariance $HP_{q,t}H^{T}+R_{q,t}$, $D\left(y_{m,t},{\hat{y}}_{q,t}\right)$ is the Mahalanobis distance between $y_{m,t}$ and ${\hat{y}}_{q,t}$, log|X| is the natural logarithm of the determinant of *X*, and $\gamma_{g}$ is a gating threshold that reduces unnecessary computations; it selects detections that are close to predictions. In this work, we solve the GNN assignment problem in Equation (9) using the optimal Munkres algorithm [47,48].

The GNN maintains its track record as follows [46]:

- Initiation: create new tentative tracks for unassigned detections; M>Q.
- Promotion: confirm a tentative track if its likelihood of being true is greater than $\gamma_{c}$.
- Demotion: demote a confirmed track to tentative if the subject leaves the ROI.
- Deletion: delete a confirmed track if its maximum likelihood decreases by $\gamma_{d}$.

Figure 4b and Figure 4f present the smoothed/tracked ground positions in the image–pixel and real-world coordinates, respectively. In addition, we overlay the plots with the original localization results in Figure 4a and Figure 4e to visualize the role of smoothing and tracking. By examining the results, one notes that the KF corrects the predicted position in gray and makes it closer to the subject’s actual location. In addition, the fifth subject’s unresolved position, because of missing information, is now compensated for by GNN—see the predicted position in blue. In summary, the smoothing and tracking stage lowers the localization error through the KF and corrects for the missing measurements by GNN. Note that this stage preserves privacy and it is intended for data correction rather than conventional tracking; hence, we are not concerned with the re-identification problem nor the subjects’ particular identities.

### 3.4. Parameter Estimation

The crowd state, in terms of social distancing behavior and congestion, is estimated by computing the inter-personal distances and the occupancy/crowd density maps.

#### 3.4.1. Inter-Personal Distance

The instantaneous pair-wise Euclidean distance between subjects *i* and *j* is expressed as:

(12) $$d_{i,j,t}=\sqrt{{\left(x_{i,t}-x_{j,t}\right)}^{2}+{\left(y_{i,t}-y_{j,t}\right)}^{2}}\;.$$

Given a social safety distance *r*, the instantaneous number of violations is computed by:

(13) $$V_{t}=\sum_{i=1}^{{\hat{N}}_{t}}\sum_{j=i+1}^{{\hat{N}}_{t}}v_{i,j,t}\;,$$

(14) $$v_{i,j,t}=\left\{\begin{array}{cc} 1 & :d_{i,j,t}\leq r\\ 0 & :d_{i,j,t}>r \end{array}\right.\;,$$

where ${\hat{N}}_{t}$ is the number of estimated/tracked people in frame *t* and $V_{t}$ counts the number of subjects that are *r* or less apart from each other—see Figure 4d and Figure 4h.

#### 3.4.2. Occupancy and Crowd Density Maps

The occupancy density map (ODM) encodes the spatial patterns exerted by the subjects in the surveilled environment [6]. It is formed by summing and averaging Gaussian functions centered at the subjects’ ground positions, i.e.:

(15) $$O\left(x,y\right)=\frac{1}{T}\int_{1}^{T}\frac{1}{{\hat{N}}_{t}}\sum_{i=1}^{{\hat{N}}_{t}}G\left(x-x_{i,t},y-y_{i,t}\right)\;dt\;,$$

(16) $$G\left(x,y\right)=\sqrt{\frac{2}{\pi \;\delta^{2}}}\exp\left(\frac{x^{2}+y^{2}}{-\delta^{2}/2}\right)\;,$$

where O(x,y) is the averaged ODM, *T* is the current frame number (or total number of frames), G(x,y) is a 2D symmetric Gaussian function, and δ controls the spatial resolution of the map. Similarly, the crowd density map (CDM) offers a spatial signature for the social distance infringements in the scene [35]. It is formulated by imposing the safety distance constraint as follows:

(17) $$C\left(x,y\right)=\frac{1}{T}\int_{1}^{T}\frac{1}{{\hat{N}}_{t}}\sum_{i=1}^{{\hat{N}}_{t}}\psi_{i,t}\;G\left(x-x_{i,t},y-y_{i,t}\right)\;dt\;,$$

where C(x,y) is the averaged CDM and $\psi_{i,t}$ is a binary mask that is 1 or 0 if subject *i* violates or follows the social safety distance *r*, respectively.

Figure 4c and Figure 4g show the instantaneous ODM in the image–pixel and real-world coordinates, respectively. In addition, we superimpose the smoothed/tracked localization results and the computed inter-personal distances in both domains. Moreover, Figure 4d and Figure 4h illustrate the instantaneous CDM in the image–pixel and real-world coordinates, respectively.

### 3.5. Anomaly Recognition

We define an irregularity in the surveillance video by the presence of social distance infractions and overcrowded, or congested, regions. We treat the first task as a classification problem by forming the binary label $S_{t}$ as follows:

(18) $$S_{t}=\left\{\begin{array}{cc} 1 & :V_{t}>0\\ 0 & :\mathrm{otherwise} \end{array}\right.\;.$$

Moreover, we consider the second task as a segmentation problem where we identify overcrowded areas in the scene by thresholding the averaged CDM as follows:

(19) $$R\left(x,y\right)=\left\{\begin{array}{cc} 1 & :C\left(x,y\right)\geq \gamma_{m}\\ 0 & :\mathrm{otherwise} \end{array}\right.\;,$$

where $\gamma_{m}$ is selected to keep 50% of the energy in C(x,y).

### 3.6. Performance Evaluation

The social distance estimation and crowd monitoring system is evaluated in terms of its ability to detect human subjects, localize their positions, recognize social distance violations, estimate crowd density maps, and to identify overcrowded regions in surveillance videos.

Let $N_{t}$ and ${\hat{N}}_{t}$ be the true and estimated/tracked number of people in frame *t*. The averaged person detection rate (PDR) and localization relative error are calculated as follows:

(20) $$\mathrm{PDR}=1-\frac{1}{T}\int_{1}^{T}\frac{\left|N_{t}-{\hat{N}}_{t}\right|}{N_{t}+1}\;dt\;,$$

(21) $$\mathrm{Error}=\frac{1}{T}\int_{1}^{T}\frac{\sqrt{{\left(x_{i,t}-{\hat{x}}_{i,t}\right)}^{2}+{\left(y_{i,t}-{\hat{y}}_{i,t}\right)}^{2}}}{\sqrt{x_{i,t}^{2}+y_{i,t}^{2}}}+\eta_{t}\;dt\;,$$

(22) $$\eta_{t}=\left\{\begin{array}{cc} \;N_{t} & :{\hat{N}}_{t}=0\\ \;{\hat{N}}_{t} & :N_{t}=0\\ |N_{t}-{\hat{N}}_{t}|/N_{t} & :\mathrm{otherwise} \end{array}\right.\;,$$

where $\left(x_{i,t},y_{i,t}\right)$ and $\left({\hat{x}}_{i,t},{\hat{y}}_{i,t}\right)$ are the true and estimated ground coordinates for subject *i* at frame *t*, respectively. We associate the estimated positions with their true counterparts using the optimal Munkres algorithm [47,48]. Moreover, given the true and predicted binary outputs $S_{t}$ and ${\hat{S}}_{t}$, respectively, we assess the detection of social distance violations by accuracy, precision, recall, and the F1-score, i.e.:

(23) $$\mathrm{Accuracy}=\frac{TP+TN}{TP+TN+FP+FN}\;,$$

(24) $$\mathrm{Precision}=\frac{TP}{TP+FP}\;,$$

(25) $$\mathrm{Recall}=\frac{TP}{TP+FN}\;,$$

(26) $$\mathrm{F1-score}=2\times \frac{\mathrm{Precision}\times \mathrm{Recall}}{\mathrm{Precision}+\mathrm{Recall}}\;,$$

where TP, TN, FP, and FN are true positives, true negatives, false positives, and false negatives, respectively. Furthermore, we complement the former evaluations by computing the averaged violations count rate (VCR), i.e.:

(27) $$\mathrm{VCR}=1-\frac{1}{T}\int_{1}^{T}\frac{\left|V_{t}-{\hat{V}}_{t}\right|}{V_{t}+1}\;dt\;,$$

where $V_{t}$ and ${\hat{V}}_{t}$ are the true and predicted counts, respectively—see Equation (13).

Finally, we evaluate the quality of the averaged CDM by the Pearson’s correlation coefficient (CORR) and assess the identified overcrowded regions using the intersection over union (IOU), i.e.:

(28) $$\mathrm{IOU}=\frac{\;\int \int \;R\left(x,y\right)\cap \hat{R}\left(x,y\right)\;dx\;dy}{\;\int \int \;R\left(x,y\right)\cup \hat{R}\left(x,y\right)\;dx\;dy}\;,$$

where R(x,y) and $\hat{R}\left(x,y\right)$ are the true and predicted thresholded averaged CDM, respectively—see Equation (19).

## 4. Results and Discussions

### 4.1. Dataset

We utilize the EPFL-MPV, EPFL-Wildtrack, and OxTown public datasets along with the pose estimations prepared in [26]. The EPFL-MPV is comprised of four sequences, named 6p-c0, 6p-c1, 6p-c2, and 6p-c3, for six people moving freely in a room [49]. The sequences are synchronized and view the same environment but from different perspectives. Each sequence is recorded at 25 frames per second (fps) and has 2954 frames. The EPFL-Wildtrack contains seven synchronized sequences, named C1-C7, with approximately 20 people moving outdoor [50]. The sequences view walking pedestrians outside the main building of the ETH university in Switzerland. They are shot using seven cameras positioned at different locations and each has a total number of 400 frames. Lastly, the OxTown is a street surveillance video with 4501 frames shot with a single camera at 25 fps. It oversees, on average, 16 people walking down a street in Oxford, London [51].

### 4.2. Preprocessing and Settings

The utilized datasets offer annotations in terms of bounding boxes that localize people in the scene. Additionally, they provide the homography matrix and the image-to-real distance scale of each recording camera. The EPFL-MPV and OxTown bounding boxes are vertically over-sized and enclose more than the areas occupied by the human subjects. Therefore, their bottom mid-points are lower than the subjects actual ground positions. In this work, we correct for this by shifting the mid-points up a percentage of the bounding box total height. In specific, we apply a 10% and 2% uplift to the EPFL-MPV and OxTown localization data, respectively. Moreover, the OxTown dataset annotation includes bounding boxes for babies in strollers/prams accompanied by adults. This is outside the scope of our work; hence, we discard them (This corresponds to the following subject IDs: 24, 42, 44, 45, and 47). Finally, the ROI for each dataset/sequence is manually selected, in the image–pixel domain, to cover the floor of the scene. The ROIs include most annotated positions, but we discard the remaining few that are outside the selected area. This corresponds to excluding 2.38% (960 out of 40,393), 6.67% (4767 out of 71,460) and 15% (6403 out of 42,721) of the EPFL-MPV, EPFL-Wildtrack, and OxTown annotations, respectively. The proposed system smoothing and tracking parameters are found for every dataset/sequence by minimizing the localization error in Equation (21) using the Bayesian optimization algorithm in MATLAB; see Table 1. The optimization is executed for 500 iterations using the expected improvement plus acquisition function and repeated five times for verification [52].

### 4.3. System Integration

**Figure 6:**
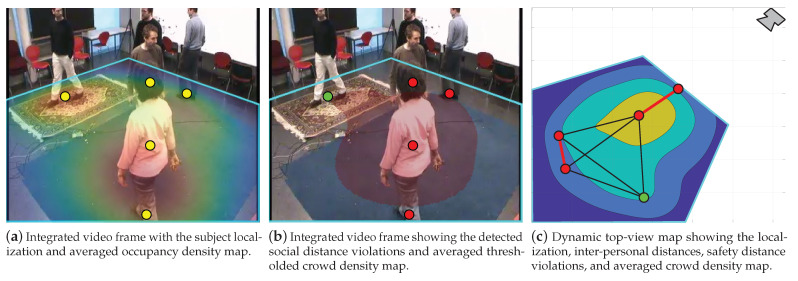
The proposed system example integrated video frames and dynamic top-view maps using frames 1 to 1824 of the EPFL-MPV dataset scene 6p-c0. The type and amount of displayed information is adjustable and one can view multiple integrated frames and/or top-view maps simultaneously. Note that the pair-wise lines in (**c**) are plotted only for distances between 0 and 3 m to ease visualization.

Figure 6 illustrates three examples for integrating the proposed system outputs and displaying them on the user interface unit. These examples offer complementary interpretations for the scene and serve different purposes depending on the intended application or required analysis. For instance, in Figure 6a, the input video frame, depicted in Figure 4, is overlaid with the localization and averaged ODM results. This type of display is important when monitoring crowds in public areas or for analyzing customer’s browsing habits and preferences in shops. Moreover, we show in Figure 6b that the former information can be replaced with the detected social distance violations and the averaged thresholded CDM. This example is directly intended for social distance monitoring applications and can be used to oversee critical waiting areas, e.g., in airports and hospitals. Furthermore, Figure 6c demonstrates a dynamic top-view map for the scene by plotting the localization, inter-personal distances, and the averaged CDM in the real-world coordinates. This figure serves as a footprint for redesigning congested areas and facilitates developing physical interaction protocols and guidelines. Finally, apart from these applications, one can merge and/or adjust the type and amount of displayed information. In addition, the user is able to view one or multiple integrated frames, or top-view maps, simultaneously; hence, offering valuable information about the scene and crowd state. The supplementary material of this paper includes videos of the system integration outcome for other video sequences.

### 4.4. Evaluations and Results

**Figure 7:**
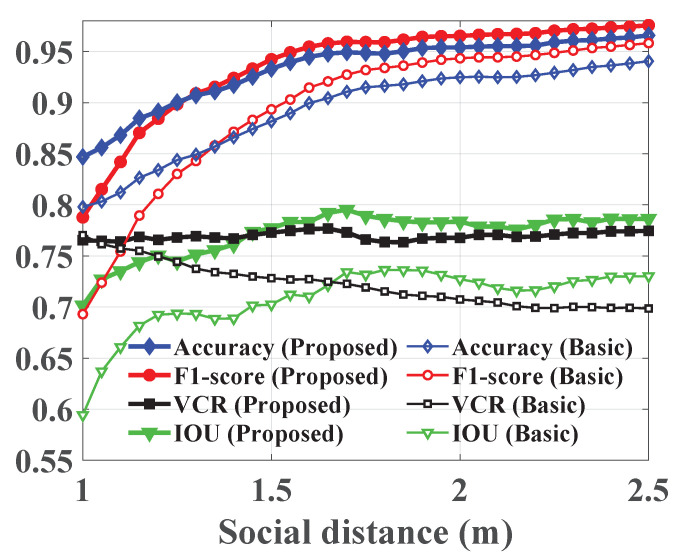
The performance evaluation results in terms of accuracy, F1-score, VCR, and IOU averaged across all video sequences and plotted for a range of social safety distances.

Figure 7 demonstrates the social distance violation detection performance of the basic and proposed approaches in terms of accuracy, F1-score, and VCR. In addition, it shows their IOU for identifying the overcrowded regions in the scene. The results are computed for a range of safety distances and averaged across all video sequences. We vary the safety distance from 1 to 2.5 m with a 0.05 step to cover a wide range of guidelines. Moreover, Table 2 illustrates the system capacity to detect human subjects, localize their positions, recognize social distance violations, estimate crowd density maps, and identify high-risk areas in each video sequence; it summarizes the PDR, localization error, accuracy, F1-score, precision, recall, VCR, CORR, and IOU. The results are averaged across the range of safety distances and we assess the gain in performance delivered by the smoothing and tracking stage.

The trends in Figure 7 indicate that the accuracy, F1-score, and IOU increase with the safety distance, whereas the VCR is stable for the proposed approach and decreases for the basic method. Additionally, they depict the gain in performance delivered by the proposed system. Specifically, the boost in accuracy, F1-score, VCR, and IOU is up to 5.8%, 9.5%, 7.6%, and 10.7%, respectively. Furthermore, by examining the results in Table 2, one notes a clear advantage for utilizing the proposed system as it yields the best overall performance across all measures, except precision to ensure balanced precision/recall trade-off. In specific, it offers the highest person detection rates and lowest localization errors for all video sequences with gains up to 43% and 38.3%, respectively. Similarly, it results in better social distance violation recognition and raises the conventional method accuracy, F1-score, and VCR by 17%, 9.6%, and 39%, respectively. Moreover, the quality of the estimated crowd density maps, in terms of correlation, is high for both techniques, because the contribution of faulty detections is insignificant to the long-term averaged estimation. However, it is not the case when identifying high-risk regions. The results highlight a growth in the IOU of the proposed method up to 12.4%; hence, it is more reliable. Finally, Table 2 emphasizes the smoothing and tracking role where it offers a considerable improvement due to its treatment for occlusions and missing data. In particular, it balances the system efficacy, by reducing the difference between precision and recall, and expands its functionality to cover various tasks and application domains.

**Figure 8:**
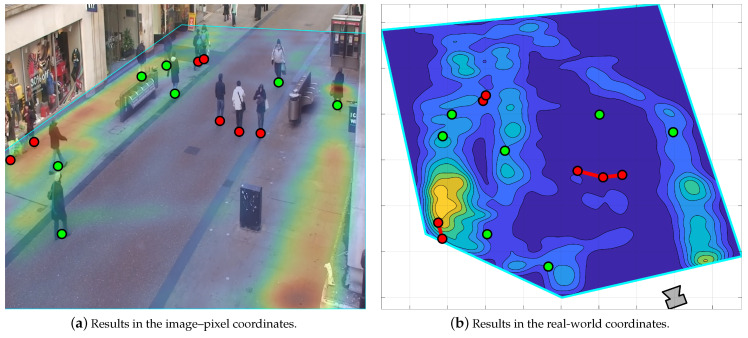
Example social distance violation detection results using frames 1 to 2005 of the OxTown dataset with a 2 m social safety distance. (**a**,**b**) overlay the detection results with the averaged ODM and CDM, respectively.

Table 3 shows a comparison between the proposed system, the basic pose-based approach from [26], and an object detection-based system developed in [15]. The comparison focuses on the systems’ ability to detect social distance violations in the OxTown dataset with a 2 m social safety distance. Note that since the compared solutions do not utilize tracking, we demonstrate the proposed system results with and without the smoothing/tracking stage. In addition, we illustrate example results in Figure 8 to visualize the proposed system outcomes. The results in Table 3 verify the proposed system applicability and the adequacy of pose-based techniques to detect social distance infractions. They indicate a 4.6% and 3% gain in accuracy and F1-score, respectively, when compared to the object detection-based method in [15]. In addition, they affirm the smoothing and tracking stage role which pushes the proposed system accuracy and F1-score by 0.9% and 0.5%, respectively.

### 4.5. Computational Complexity Analysis

**Figure 9:**
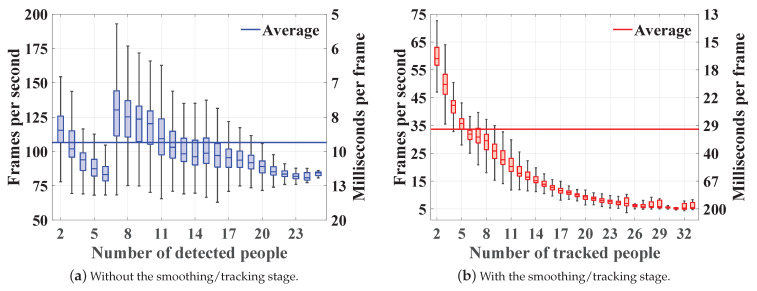
The computational complexity analysis results in terms of frame and processing rates. The proposed approach is tested with and without the smoothing/tracking stage. The results are grouped by the number of detected/tracked subjects.

The complexity of the proposed system is measured by its frame rate; the number of processed video frames per second, and processing rate; the amount of processing time per frame. The assessment is conducted by Monte-Carlo simulations where we run the model depicted in Figure 1 using all video frames and repeat the process ten times for validation. Note that we exclude the complexity of OpenPose since we use the pre-computed poses in [26]. Nevertheless, OpenPose real-time operation on both CPU and GPU machines was verified in [37,53]. In addition, we select OpenPose due to its simplicity and availability, but it can be replaced with any other pose estimation model given the same body joints indexing scheme described in Section 3.1.1. We use a desktop equipped with 2 Intel${}^{®}$ Xeon${}^{®}$ E5-2697V2 x64-based processors, 192 GB of memory, and MATLAB R2020b. Figure 9 demonstrates the developed system frame and processing rates with respect to the number of detected/tracked subjects. The averaged results suggest that the system is capable of running in real-time despite the smoothing/tracking stage additional complexity. Specifically, it runs at 106.5 fps (9.9 ms/frame) when solely relying on the proposed localization strategy and at 33.6 fps (44.5 ms/frame) when accommodating the tracking algorithm. Moreover, the results indicate that the localization approach complexity depends on the amount of occlusions present in the video frame—see Figure 9a. This is shown by the drop in frame rate when 2–6 people are present and by its slow decline when having more than 7 people in the scene. The first drop is caused by the EPFL-MPV dataset where we have six subjects moving in a highly confined environment resulting in many occlusions, while the second is due to the general increase in the number of people, which escalates the chances of occlusion. Furthermore, the smoothing/tracking introduced complexity is demonstrated by the frame rate rapid decay when increasing the number of subjects—see Figure 9b. The trends reveal the system limited ability to resolve highly dense crowds. In particular, the average frame rate drops below 25 fps (40 ms/frame) and 12 fps (83 ms/frame) when we have more than 10 and 17 people, respectively. Nevertheless, these findings highlight a need to distribute the computational load across the surveillance infrastructure. For instance, stages 1–4 in Figure 1 can be performed locally by the camera or on edge devices, while stages 5–9 require more resources.

## 5. Conclusions

The COVID-19 pandemic has deemed social distancing a critical first line of defense against its wide spread; nevertheless, safety distance guidelines are not always followed. Monitoring social distancing is important to draw realistic mitigation plans and to structure exit strategies. However, it is a labor-intensive task and suffers from subjective interpretations; therefore, combining computer vision and machine learning models with mass surveillance is intuitive for automation, but it must preserve privacy to ensure ethical adoption and application.

This work presented a privacy-preserving adaptive social distance estimation and crowd monitoring system for surveillance cameras. We evaluated the system’s ability to detect human subjects, localize their positions, recognize social distance violations, estimate crowd density maps, and identify high-risk areas. Additionally, we analyzed its computational complexity in terms of processing time. The results indicated a clear advantage for utilizing the proposed localization approach when compared to the latest techniques. In addition, they showed a considerable improvement delivered by the adaptive smoothing and tracking stage. Specifically, the system improves the PDR, localization relative error, accuracy, F1-score, VCR, and IOU up to 43%, 38.3%, 17%, 9.6%, 39%, and 12.4%, respectively. In addition, it runs at 33.6 fps (44.5 ms/frame) making it a real-time solution for low to medium-dense crowds. The proposed system occupancy/crowd density map functionality extends its application domain beyond the COVID-19 pandemic to cover other areas. For instance, it can help re-configure or re-design common physical layouts and relocate facilities in businesses to optimally reduce congestion. Additionally, it is capable of facilitating the analysis of customer’s browsing habits in shops and quantifying the effectiveness of marketing kiosks.

The developed system, although advantageous, is still limited and can be extended in various ways such as: (1) estimating the body orientation to relax the assumption of vertically oriented subjects; (2) fuse detections and estimations from multi-view cameras to assess the environment state rather than the camera specific scenery; (3) develop an automatic online training paradigm for the tracking algorithm parameters; (4) embed regression techniques to estimate the crowd density maps; (5) detect other anomalies such as fire, smoke, unattended objects in public places, and abnormal individual or crowd behavior. These will be the topics of our future research. 

## Figures and Tables

**Table 1:** The proposed system smoothing and tracking optimized parameters for each utilized video sequence.

Sequence	Parameters					
	$\sigma_{1}$	$\sigma_{2}$	$\sigma_{3}$	$\gamma_{g}$	$\gamma_{c}$	$\gamma_{d}$
**6p-c0**	6 $\times \;10^{-9}$	0.204	2.01	398	76	−186
**6p-c1**	8.21	0.329	8.78	399	64	−71
**6p-c2**	0.216	0.278	0.271	52	52	−84
**6p-c3**	7 $\times \;10^{3}$	0.015	1.76	104	86	−106
**OxTown**	2 $\times \;10^{-4}$	0.873	0.142	23	11	−26
**C1**	7 $\times \;10^{-9}$	6 $\times \;10^{-9}$	2 $\times \;10^{-5}$	11	1	−92
**C2**	0.002	$10^{-9}$	1.46	12	8	−181
**C3**	0.02	0.0078	6 $\times \;10^{-8}$	139	2	−5
**C4**	429	0.022	0.062	81	3	−2
**C5**	$10^{-8}$	2.05	3.79	15	3	−41
**C6**	9 $\times \;10^{3}$	$10^{-7}$	4 $\times \;10^{-8}$	15	9	−106
**C7**	5 $\times \;10^{-7}$	0.0006	4 $\times \;10^{-7}$	395	1	−2

**Table 2:** The performance evaluation results in terms of PDR, localization relative error, accuracy, F1-score, precision, recall, VCR, CORR, and IOU averaged across the range of safety distances and summarized for each video sequence. The proposed approach is evaluated with (✓) and without (✕) the smoothing/tracking stage (S/T). Best results are in bold to ease interpretation and results depicting highest gain are in brackets for comparison.

Measure	Approach	S/T	EPFL-MPV				OxTown	EPFL-Wildtrack							Overall
			6p-c0	6p-c1	6p-c2	6p-c3		C1	C2	C3	C4	C5	C6	C7	
**PDR**	Basic [26]	-	90.9	90.7	87.5	87.3	85.7	59.2	56.6	74.2	87.3	78.2	(39.9)	91.3	85.4
	Proposed	✕	93.8	94.0	89.1	88.1	88.4	64.7	62.6	79.1	**88.5**	80.8	43.5	92.5	87.9
		✓	**95.6**	**96.5**	**91.9**	**90.8**	**89.8**	**84.4**	**83.4**	**79.2**	**88.5**	**83.9**	(**82.9**)	**92.6**	**91.5**
**Error**	Basic [26]	-	17.0	17.2	23.0	21.9	24.0	51.3	52.8	49.0	41.3	33.8	(80.4)	15.4	24.7
	Proposed	✕	12.6	13.2	20.7	20.6	20.8	47.0	47.1	49.8	39.8	30.9	76.1	14.3	21.7
		✓	**10.7**	**10.7**	**16.6**	**17.8**	**19.1**	**31.9**	**36.7**	**48.3**	**36.3**	**27.0**	(**42.0**)	**14.0**	**18.0**
**Accuracy**	Basic [26]	-	91.0	89.2	83.3	85.6	92.8	95.5	97.7	92.5	88.0	92.3	(82.7)	**97.0**	89.3
	Proposed	✕	94.1	92.7	86.5	88.2	94.5	96.5	98.6	**93.5**	**88.5**	**93.0**	87.0	96.9	91.8
		✓	**94.9**	**94.6**	**88.2**	**89.8**	**95.6**	**99.4**	**99.7**	**93.5**	88.1	92.7	(**99.7**)	96.9	**93.3**
**F1-score**	Basic [26]	-	90.7	89.1	80.7	83.8	95.9	97.7	98.8	95.9	79.2	95.7	(90.2)	**98.1**	89.5
	Proposed	✕	94.4	92.9	85.0	87.5	96.9	98.2	99.3	**96.5**	**81.2**	**96.0**	92.9	**98.1**	92.3
		✓	**95.2**	**94.9**	**87.0**	**89.5**	**97.5**	**99.7**	**99.8**	**96.5**	80.0	95.9	(**99.8**)	**98.1**	**93.6**
**Precision**	Basic [26]	-	**98.2**	**98.1**	**98.4**	**95.6**	**97.4**	**100**	**100**	**98.7**	**88.8**	**100**	**100**	**99.0**	**97.6**
	Proposed	✕	96.0	97.4	96.8	93.4	97.2	**100**	**100**	97.4	85.0	99.8	**100**	98.6	96.4
		✓	95.7	97.0	96.4	91.7	97.0	**100**	**100**	97.4	86.4	98.2	**100**	98.6	95.9
**Recall**	Basic [26]	-	84.8	82.6	69.5	75.4	94.4	95.5	97.7	93.5	71.6	91.7	(82.7)	97.3	83.7
	Proposed	✕	92.8	89.3	76.5	82.6	96.6	96.5	98.6	**95.8**	**77.9**	92.6	86.9	**97.6**	89.0
		✓	**94.6**	**93.0**	**79.8**	**87.5**	**98.1**	**99.4**	**99.7**	95.7	74.7	**93.7**	(**99.7**)	**97.6**	**91.8**
**VCR**	Basic [26]	-	81.3	78.5	78.1	78.7	64.8	33.5	36.5	46.1	**86.7**	72.6	(20.9)	86.8	72.2
	Proposed	✕	84.3	83.7	80.1	**80.1**	**67.6**	38.4	43.1	**48.3**	85.9	**75.9**	26.0	**89.7**	75.1
		✓	**86.0**	**86.6**	**81.4**	79.6	65.1	**62.5**	**63.2**	47.5	86.5	73.3	(**59.8**)	**89.7**	**77.0**
**CORR**	Basic [26]	-	98.3	99.1	98.9	98.9	(85.3)	89.1	73.4	85.8	96.4	88.2	72.3	**98.8**	93.8
	Proposed	✕	99.2	**99.4**	**99.2**	**99.3**	89.4	**90.0**	72.5	86.0	96.8	90.7	72.3	98.6	95.1
		✓	**99.4**	99.2	**99.2**	99.2	(**89.9**)	89.3	**77.3**	**86.3**	**97.1**	**91.1**	**72.8**	98.6	**95.3**
**IOU**	Basic [26]	-	(74.7)	86.2	84.1	84.0	52.2	51.8	47.1	**63.8**	61.9	44.7	13.9	**83.8**	70.8
	Proposed	✕	83.4	**89.6**	85.3	**86.1**	61.0	**55.7**	51.3	61.5	66.9	47.3	14.4	83.4	75.6
		✓	(**87.1**)	89.2	**86.3**	85.7	**63.8**	55.3	**55.2**	61.6	**68.2**	**50.7**	**22.3**	83.4	**77.1**

**Table 3:** Comparison for the social distance violation detection performance using the OxTown dataset with a 2 m social safety distance. The Yang et al. results are extracted from Table 6 in [15]. The proposed approach is compared with (✓) and without (✕) the smoothing/tracking stage (S/T). Best results are in bold to ease interpretation and results that are used in the discussion are in brackets.

Method	Accuracy	F1-Score	Precision	Recall
Yang et al. [15]	(92.8)	(95.6)	95.4	95.9
Basic [26]	96.0	97.9	**98.9**	96.8
Proposed, S/T: ✕	(97.4)	(98.6)	98.8	98.4
Proposed, S/T: ✓	(**98.3**)	(**99.1**)	98.7	**99.5**

## Data Availability

Our system is open-sourced. The implementation and the experiment data can be assessed via our GitHub repository: https://github.com/Al-Sad/Social-Pose, accessed on 5 January 2022.

## Supplementary Materials

The following are available at https://github.com/Al-Sad/Social-Pose.
